# Enhanced Molten Salt Resistance by Sidewall Pores Repair during Fs Laser Drilling of a Thermal Barrier-Coated Superalloy

**DOI:** 10.3390/ma12121905

**Published:** 2019-06-13

**Authors:** Zhengjie Fan, Xiaomao Sun, Xuesong Mei, Rujia Wang

**Affiliations:** State Key Laboratory for Manufacturing Systems Engineering, Xi′an Jiaotong University, Xi′an 710049, China; m161125@stu.xjtu.edu.cn (X.S.); xsmei@xjtu.edu.cn (X.M.); jiaa1991@163.com (R.W.)

**Keywords:** laser processing, TBCs, pore repairing, thermal effect, corrosion

## Abstract

In this study, a novel laser-modified drilling method was used to manufacture cooling holes through thermal barrier coatings (TBCs). Due to the “cooling processing” properties during low-frequency femtosecond (LF-fs) laser drilling, the exposure of the sidewall pores, and the interlayer clearance, the inherent characteristics of plasma-sprayed coatings induced sidewall defects in the drilled holes. After drilling, a high-frequency fs (HF-fs) laser was used to repair the sidewall pores and interlayer clearance of the drilled ceramic holes. Then, the pores and microcracks were healed by local melting using the laser. Moreover, instead of obtaining laser-induced periodic surface structures (LIPSSs), refined and homogeneous grains were produced by the HF-fs laser repair treatment at high transient pressure and temperature. The results from a high-temperature corrosion test showed that healing of the open pores and microstructural improvement in the ceramic hole walls prevented the out-diffusion of Y_2_O_3_ stabilizers and the penetration of molten salt, resulting in less corrosive products and producing corresponding phase-transformation stress. Thus, reducing the stabilizer consumption can moderate corrosion fatigue and prolong the lifetime of a cooling hole and TBCs under service.

## 1. Introduction

Higher film-cooling hole requirements have been proposed for the new generation of aircraft engines. Efficiently processing these film-cooling holes in aircraft engines with thermal barrier coatings (TBCs) is becoming a key issue. However, drilling through a multilayer material composed of superalloys and ceramics is difficult with conventional machining methods, e.g., electrochemical machining (ECM) and electro-discharge machining (EDM), because this material is insulating, which restricts its range of applications [1]. Laser drilling is now becoming a trend in fabricating cooling holes in thermal barrier-coated turbine blades as it can drill these difficult materials with high accuracy and efficiency and it can be used with any geometry [2,3,4]. Despite the above advantages, traditional long-pulsed (millisecond and nanosecond) laser drilling still faces unavoidable imperfections such as TBCs delamination, spatter deposition, and barreling [5,6,7,8,9].

**Figure 1:**
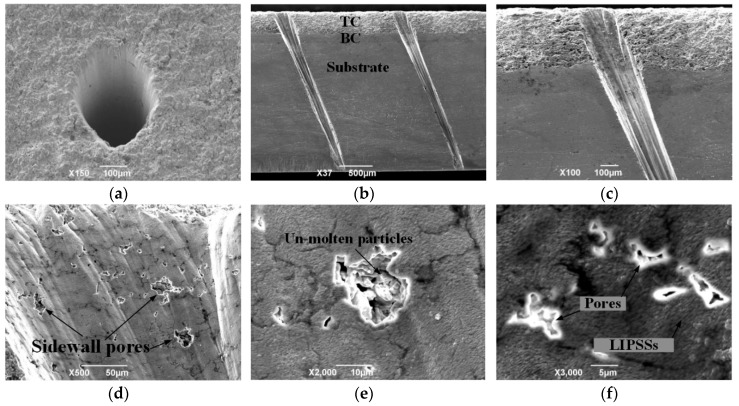
(**a**) Low-magnification image of the sample surface; (**b**,**c**) cross-sectional morphology; (**d**–**f**) walls of holes drilled with a femtosecond (fs) laser at 5 W power and 1 kHz repetition rate through a Ni superalloy with thermal barrier coating (TBC). LIPSSs: laser-induced periodic surface structures.

Ultrashort laser drilling in multilayer materials has received more attention in recent years with the gradual improvement of energy density [10,11]. Because of the ultrashort laser pulse (100 fs), the ablated materials will sublimate with limited heat diffusion and no remnant molten material [12,13]. For the fs laser-drilled holes, as shown in Figure 1a–c, delamination cracks and spatter were not observed near the machined area. Generally, in order obtain excellent properties, i.e., low thermal conductivity and Young’s modulus and high strain tolerance, atmospheric plasma spraying (APS) yttria-stabilized zirconia (YSZ) coatings have been usually produced with high porosity [14,15]. However, under the most demanding operating conditions, these porous YSZ coatings are prone to hot corrosion caused by molten salts, i.e., Na, S, and V [16,17]. This can cause a reaction between yttria and V_2_O_5_ and consumption of Y_2_O_3_ (the stabilizer of YSZ), resulting in premature failure of the TBCs [18]. In addition, certain pores are exposed to the wall of the ceramic hole section (see from the high-magnification image of the femtosecond (fs) laser-drilled hole in Figure 1d–f) resulting from the fs-laser “cooling processing” properties. These open pores will accelerate the corrosion of the molten salts. Therefore, further surface treatment is required to eliminate these micro-defects during fs laser drilling of cooling holes for applications in harsh service conditions.

Herein, we report a method for drilling cooling holes in a Ni superalloy with TBC with an fs-laser, in which the sidewall pores and interlayer microcracks can thermally heal with the high-frequency fs (HF-fs) laser. In addition, the effect of the repair of pores and cracks on the resistance of high-temperature molten salts was investigated.

## 2. Materials and Methods

A Ni superalloy (Inconel 718) with 2 mm thickness was low-temperature supersonic-flame sprayed with an intermetallic Ni–Co–Cr–Al–Y–Ta bondcoat (BC, approximately 100 μm thick) and air-plasma sprayed with a partially yttria-stabilized ZrO_2_-7 wt % Y_2_O_3_ topcoat (TC, 300–350 μm thick). A low-temperature supersonic-flame spraying system (K2, GTV, Luckenbach, Germany) equipped with an IRB2400 ABB robot and a three-anode atmospheric-plasma spraying system (APS, MF-P-1000, GTV, Luchenbach, Germany) were used to prepare the BC and TC, respectively. A neodymium-doped lithium yttrium fluoride (Nd:YLF) solid-state fs laser system (*λ* = 800 nm, Spectra-physics) with 1 kHz repetition rate, 3.6 W pulse power, and 120 fs pulse duration was used to irradiate the TBC surface at normal incidence. The trepan diameter and drilling rate were 0.5 mm and of 0.5 mm s^−1^, respectively. Following drilling, a diode-pumped fs laser (Phoras-20W, *λ* = 1030 nm, Pharos, Vilnius, Lithuania) with 100 kHz repetition rate, 1 W pulse power, 240 fs pulse duration, and 2 mm defocus (referring to the upper surface of the TBC) was used to repair the hole wall with the trepan and spiral mixed mode. The trepan diameter was 0.3 mm with a scanning rate of 5 mm/s. The laser beam was directed along the axial direction with 30 μm feed. The process was repeated 120 times.

The hot corrosion resistance of the laser-drilled holes with and without HF-fs laser repair was tested at 1050 °C for 30 h. A mixture of Na_2_SO_4_ (50 wt %) and V_2_O_5_ (50 wt %) was prepared. The salts were dissolved in distilled water to yield a 40 wt % salt solution. The salt solution was then used to fill the drilled holes. The microstructure of the manufactured holes was examined using a scanning electron microscope (SEM, JEOL, Tokyo, Japan). The quantitative phase compositions of the unrepaired and repaired ceramic holes were identified by an X-ray diffractometer (XRD, D/MAX-2400, Rigaku, Tokyo, Japan) using a Cu target Kα produced at 40 KV and 100 mA with scanning speed of one step/s and step length of 0.02°.

## 3. Results and Discussion

**Figure 2:**
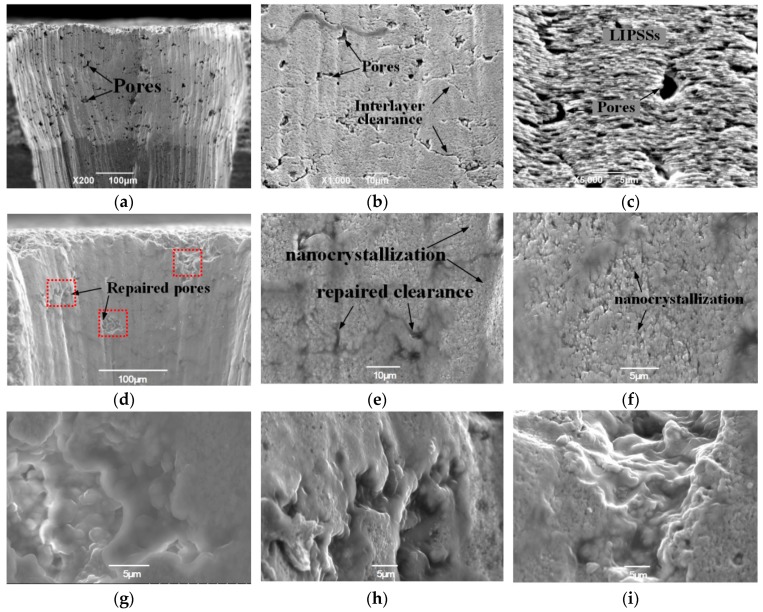
SEM images showing cross sections of low-frequency femtosecond (LF-fs) laser-drilled holes: (**a**–**c**) unrepaired, (**d**–**i**) repaired with high-frequency (HF)-fs laser, (**b**,**c**) microstructure details in (**a**), and (**e**–**i**) repaired details in (**d**).

Figure 2a–c shows the cross-sectional morphology of the holes drilled with the low-frequency (LF)-fs laser. The sizes of the open pores ranged from approximately from 2 to 20 μm, and the pores were located at the side wall of the drilled ceramic holes. Noticeably, many TBC interlayer clearances in the as-sprayed coatings can be seen in the magnified SEM image in Figure 2b. Moreover, laser-induced periodic surface structures (LIPSSs), resulting from the interference between the incident laser beam and the excited surface electromagnetic wave [19,20], are also evident in Figure 2c. Figure 2d–i shows an overview of the microstructure and detailed microstructural characteristics of holes repaired with the HF-fs laser. It appears that the open pores and interlayer microcracks were entirely healed by the HF-fs laser, as shown in Figure 2a,b. This indicates that the HF-fs laser produced thermal repair of unmolten or semi-molten ceramic particles due to intense plasma irradiation. These particles, presumably, arose more likely in the porous zone if the HF-fs laser was off-focus, inducing thermal accumulation due to multiple reflections. Figure 2c shows that the repair treatment at high transient pressure and temperature produced a refined, homogeneous hole wall microstructure (average grain size approximately 200 nm~500 nm). The mechanism of the interaction of the HF-fs with ceramic is very complex, and the microstructure evolution is determined by many factors. One of the most important factors is represented by laser-induced surface plasmons. It is said that atomic-scale ion–matter interactions and mass transport can be controlled by ion-beam flux during irradiation, which can enable to shape the nanoscale structure [21]. Similarly, the HF-fs can significantly increase the surface plasmons flux nearby the irradiated surface and accordingly develop surface nanocrystallization and refined microstructure. However, the mechanism of the HF-fs interaction with ceramic and the microstructure evolution need further study. It can be seen from the microstructure of the representative repaired open pores (Figure 2g–i) that unmelted ceramic particles melted and eliminated any clearance between them. It is noteworthy that the melt only existed in these loose porous zones, and multiple internal reflections may be more prone to occur among the un-melted particles. Thus, the HF-fs laser is useful for repairing microcracks and pores because of its thermal effect in the suppressed-heat affected zone.

**Figure 3:**
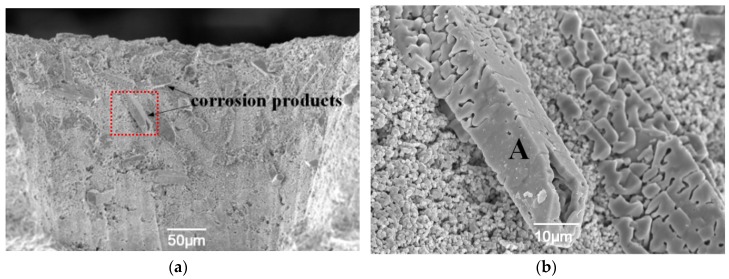

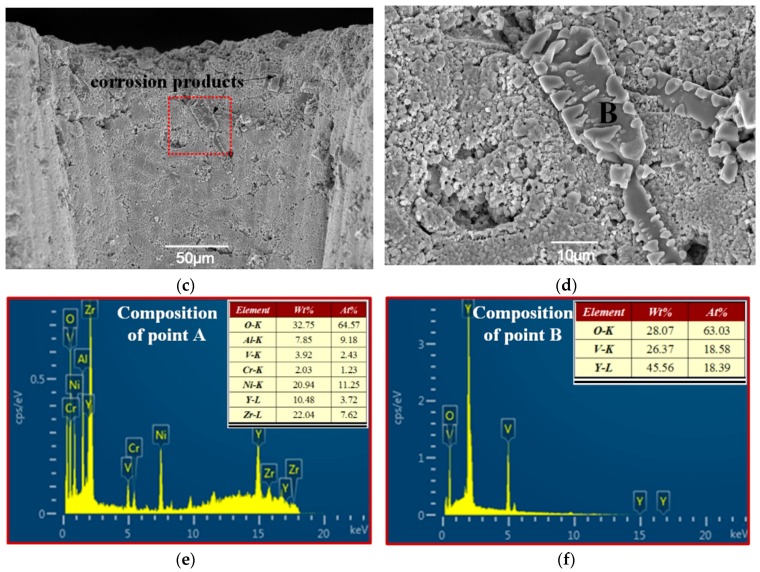
Cross-sectional morphology of the drilled holes: (**a**,**b**) unrepaired, (**c**,**d**) repaired with HF-fs laser after exposure to Na_2_SO_4_ + V_2_O_5_ at 1050 °C for 30 h, (**e**,**f**) EDS results from points A and B, respectively.

Figure 3 shows the cross-sectional morphology of unrepaired and repaired cooling holes after exposure to molten salt (Na_2_SO_4_ + V_2_O_5_) at 1050 °C for 30 h, where corrosion products (crystals) were deposited on the hole wall. The unrepaired samples show enormous quadrilateral prism-shaped crystals (Figure 3a,b), many with sizes greater than 50 μm, indicating severe corrosion. Some corrosion products grew out of the open pores and filled the gap. However, only a few plate-like and thin-strip crystals were found in the repaired holes (Figure 3c,d). Compared to the unrepaired holes, these crystals were fewer and smaller in the repaired holes. The healed pores had not been attacked by the molten salts. The unrepaired pores and interlayer clearance provided a diffusion path for the molten salts to penetrate into the YSZ coating and for the stabilizer (Y_2_O_3_) to precipitate from the YSZ coating and diffuse outward the hole wall. This process involved the following reactions [22,23]:

(1) $$V_{2}O_{5}\;\left(s\right)+Na_{2}SO_{4}\;\left(l\right)\to \;NaVO_{3}\;\left(l\right)+SO_{3}\;\left(g\right)$$

(2) $$ZrO_{2}\cdot Y_{2}O_{3}\;\left(s\right)+2NaVO_{3}\;\left(l\right)\to m-ZrO_{2}\;\left(s\right)+2YVO_{4}\;\left(s\right)+Na_{2}O\;\left(l\right)$$

**Figure 4:**
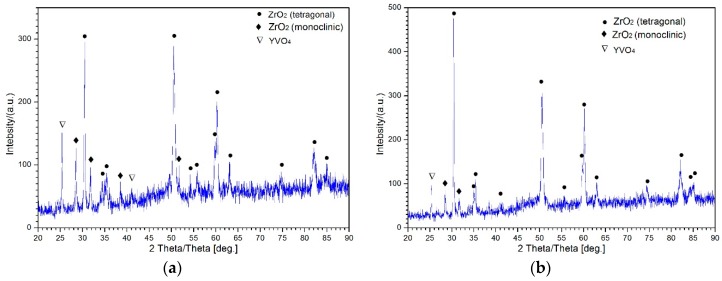
XRD patterns of ceramic hole walls: (**a**) unrepaired, (**b**) repaired with an HF-fs laser after hot corrosion.

The molten salts would subsequently react with Y_2_O_3_ to form YVO_4_ crystals, resulting in depletion of the stabilizer, and trigger the subsequent phase transformation from tetragonal to monoclinic, leading to YSZ sintering [24]. The undesirable phase transformation from tetragonal to monoclinic was accompanied by a 3–5% volume expansion, which led to phase transformation stress in the coatings. Consequently, the growth stress of the YVO_4_ crystals and the phase transformation stress collectively caused premature failure. EDS (Figure 3e) and XRD analyses (Figure 4a) confirmed that the corrosion products were mainly composed of YVO_4_ for the unrepaired holes. Other than yttrium and vanadium, the EDS spectrum of corrosion products (Figure 3e) contained peaks corresponding to some fundamental elements (i.e., zirconium, nickel, chromium, etc.); constituents of the TBC and BC were also found. One can conclude that these elements also diffused through pores or clearances and reacted with the molten salts, inducing more severe corrosion. However, the healed pores and microcracks prevented the out-diffusion of Y_2_O_3_ stabilizers and the penetration of the molten salt. Moreover, unlike the undulations in LIPSSs, the smooth surface of the microstructure and compact fine grains can effectively improve the corrosion resistance by storing less molten salts, thus decreasing the reactive area and enhancing the mechanical properties of the microstructure in the hole wall. EDS analysis of the crystal revealed that the plate-like corrosion product was composed of yttrium, vanadium, and oxygen (see Figure 3f). Further XRD analysis confirmed that the corrosion products consisted of YVO_4_ (Figure 4b). This is in accordance with the results obtained in previous researches [25]. Moreover, the XRD patterns showed that, after hot corrosion, all samples contained tetragonal and monoclinic phases, while the diffraction peaks of monoclinic phases and YVO_4_ had greater intensity in the corresponding XRD patterns of the unrepaired samples (Figure 4a) compared to the repaired samples (Figure 4b). These results further confirmed that the unrepaired cooling holes suffered from the serious corrosion damage.

## 4. Conclusions

An HF-fs laser was used to repair open pores and interlayer clearances in a TBC on an Ni superalloy in order to improve their molten salt resistance. It is very interesting that the HF-fs had both an inductive effect and a thermal effect. In porous and uneven areas, the HF-fs was more prone to induce a thermal accumulation effect, which was likely be due to multiple reflection and volume absorption, accordingly melting the unmolten particles and healing the pores. Moreover, the HF-fs could induce intensive plasma in the processing area, which might affect the melting of the unmolten particles. In the smooth section of the ceramic holes, the HF-fs tended to exhibit the inductive characteristic of plasma exciton. That is, the HF-fs laser produced local high transient pressure and temperature nearby the irradiated surface, refining the microstructure and inducing surface nanocrystallization. However, the mechanism of HF-fs interaction with ceramic is very complex and needs to be investigated further. The corrosion test results showed that the repaired pores could effectively prevent the out-diffusion of Y_2_O_3_ stabilizers and the penetration of molten salt. Besides, the repaired and refined hole walls were flatter and provided less room for salt deposit nucleation, resulting in less salt crystals inside the holes. Thus, the repaired holes could effectively enhance the molten salt resistance and prolong the service life of the TBCs.
